# Excision of Elbow-Joint

**Published:** 1856-07

**Authors:** 


*Excision of the Elbow-Joint*; *Recovery; the motions of the Forearm and Hand, with the functions of the limb, preserved.* By Richard G. H. Butcher, Surgeon to Mercer's Hospital.

When a case of incurable disease of the elbow-joint presents itself to the surgeon, the first question for consideration is one upon which I have laid much stress when treating of excision of the knee — namely, is the case one which must be submitted to amputation, or is it one to which resection is applicable? Now if this distinction be not made, if a painstaking investigation be not instituted, an error in diagnosis may do one or two things—either the wretched sufferer may be doomed to mutilation for life, or resection (a most valuable operation) may be brought into discredit by its performance where not at all suited, and where it was never intended by its warmest supporters. Again, it should ever be remembered that a patient may not have the power to bear amputation, after failure by an ill-advised resection, when he might have made a good recovery after amputation, if performed at first.

An all-important point to establish, is, to what extent are the bones engaged? Through fistulous openings the surgeon, almost invariably, will have the power of determining with the probe and by manipulation, according to his tact, the amount of disease; the entire carious part must be cut away — therefore, when extending much beyond the articular surfaces, resection should not be attempted, as it is plain, removal of a few inches of each bone could not be followed by even an approximation to cure in any way satisfactory. Here, then, amputation takes its proper place. A wide distinction, however, must be drawn between the hardened, roughened surface of the shafts of the bones in the immediate vicinity of carious joints, and the carious surfaces themselves which in most instances are confined merely to the articulating extremities. I wish to lay stress upon this vital distinction, because men have conveyed by their writings and delineations (though never intending to do so) the consummation of this blunder. In some instances, no doubt, some of these roughened spiculæ must be pared off, to permit the parts to come evenly in contact; but this is a very different thing from cutting away three or four inches from the humerus. There is this distinction between the two conditions: the one is the result of excessive action, as it were limiting disease, the other is the disease established and progressive. Now the thickened integuments, apparently disorganized, pierced with jagged apertures, through which escape the unhealthy secretions from the vicinity of the diseased bones within, need form no obstacle to resection; though livid, dark, and unhealthy looking, it is marvelous in how short a time they recover when the sources of irritation have been taken away. The gelatinous infiltrated tissues become softened down by suppuration, and are cast off and replaced by a more healthy and vital action, that of granulation, the precursor of cicatrization and cure. The age of the patient in a secondary way, may be mentioned in considering the question of resection of the elbow; however, I think the surgeon will be best able to form an opinion according to the individual case. I have seen some in advanced age repair injuries, and go through operations with as persistent a recovery as could be desired, when liberally supplied with stimulants. No doubt the plastic efforts of nature will be more marked and her restorative energy more decided during the elasticity and vigor of youth; yet if gently and carefully assisted by the surgeon, she will not abandon the young or the old; however, in young subjects sometimes the scrofulous taint, poisoning, as it were, every healthy function, restlessly active, may create an insuperable obstacle to resection.

The annexed case offers practically an illustration of that which I conceive to be the best mode of performing the operation, and of conducting it to a favorable issue.

J. F., aged 56, admitted to Mercer's Hospital, July 8, 1854. *History.*—He had been a soldier, and served for many years, exposed to several vicissitudes and extremes of climate; on four occasions he suffered from syphilis, prolonged with its secondary and tertiary forms, but each yielded to treatment. The patient associated the commencement of his present sufferings with a severe wrench of the right elbow-joint, which originated in a scuffle with a tipsy companion eight years before, and during this lengthened period pain was never absent, though at times but trifling, yet at others almost unbearable. The joint was not only injured and violently disruptured, but the soft parts all around were extensively ecchymosed from contusion, the man having fallen upon him. Various modes of treatment, those usually adopted in similar cases, were had recourse to, but with little benefit and only temporary ease; latterly his annoyances became so much more permanent, that his health was entirely broken up, and the constitutional derangement, as a sequel to sleepless nights, loss of appetite, and perpetual restlessness, made him seek for advice in hospital. When received under my charge on the above date his case was painfully typical of the prostrating effects, the corroding results of a diseased joint, paralyzing, as it were, the powers of vitality, and vitiating the very springs of life; the patient was sadly reduced; from being an athletic man, he was thin, emaciated, and haggard; loss of appetite, occasional diarrhœa, night-sweats, scanty renal secretions, evidenced the disturbance of the digestive functions; listlessness, weakness, and an inability to exertion, predominated over the muscular system; while pain, ever varying in acuteness and duration, hallucinations in imperfect sleep, partial stupefaction, sometimes from intensity of suffering, ending in total apathy as to recovery, evidenced how deeply the nervous system sympathized in the local malady. The changes wrought in the limb were strikingly characteristic of long-continued disease; the shoulder and upper third of the arm was wasted, the forearm and hand attenuated, the fingers being extended, flattened, and the integuments covering them being thin, shining, glazed, with the unguinal phalanges slightly puffed, and nails bent.

As before mentioned, the forearm was fixed at nearly a straight line with the arm; all around the elbow-joint was inordinately swollen, the circular measurement corresponding to the flexure being 15½ inches, while the arm in its upper third only measured 6½ inches, and the forearm, at the junction of the lower and midle third, only 7 inches. The integument covering the joint and adjacent parts was in many places of a dark livid color, while immediately on the confines of the swelling it assumed a mottled appearance. Numerous apertures existed around the articulation, in all, eight in number; five were situated on the inner side of the limb, three behind the internal edge of the olecranon, and one on the outside, somewhat over the head of the radius.

The great bulk of the limb corresponding to the diseased joint was elastic, harder in some places than in others, and made up of depositions of fibrin, consequent upon the oft-repeated and violent attacks of inflammation, perpetuated for many years. Several of the sinuses presented at their apertures everted, fleshy papillæ, bleeding on the slightest touch; through them the diseased condition of the bones entering into the articulation was clearly revealed; a probe gently passed along the trajet of either, soon came in contact with the denuded carious bone. From the great thickness of the parts around the joint, and the spastic action of the muscles imposed by a rigid position, the motions of flexion and extension were prohibited; slight pronation could be effected, and grating of the head of the radius on its pivot was very audible at the time. By these various sources, and the method of examination, then, a very accurate conclusion was arrived at as to the totally disorganized condition of the joint—the decay and death of the extremities of the bones formerly constituting its beauty and perfection. From a very careful investigation and minute inquiry into all the bearings of the case, I was forcibly impressed with the conviction that the shafts of the bones were not involved; by the most pains-taking manipulation with the probe, I could only discover the condyles of the humerus, and the olecranon process, together with the head of the radius, diseased; the thickening and induration above and below the joint, I presumed, had its origin and seat in the soft parts, as the product of repeated inflammatory action. Upon the foregoing inferences I rejected amputation, and decided on excising the diseased extremities of the bones forming the articulation, and proceeded after the following manner: The patient being placed upon a table in the horizontal position, and lying on his back, chloroform was administered, and on its effects being produced the limb was drawn from the side and elevated; one assistant commanded the brachial artery, and steadied the arm, while another supported the forearm. Standing on the right side of the patient, I made an incision about an inch above the internal condyle, and a little behind the ridge leading to it, and extending downwards in a direct line to a little below the junction of the coronoid process with the body of the ulna. The wound in its entire length measured about two inches and a half; an incision of like extent was next made over the external condyle, the ridge leading to it, and the head of the radius. The internal of these incisions was so planned that it permitted the ulnar nerve to be quickly freed from its sheath behind the condyle, and drawn without much disturbance, together with the soft parts, somewhat in front of that prominence; thus this important part was protected during the formation of the third incision, which connected the lateral longitudinal ones, and lay in a transverse direction over the extremity of the olecranon. The attachment of the triceps being cut through, the upper flap was raised and held upwards, while the parts covering the olecranon, and constituting the lower flap, were rapidly dissected from the bones and restrained downwards. The lateral ligaments were next cut through, and the forearm violently flexed; and the ends of the disarticulated bones thrust backwards through the wound. Scarcely had this measure been accomplished, when the accuracy of the diagnosis was fully confirmed; the ends of the bones seemed alone to participate in the mischief, but to their condition I shall again allude. The excision of the end of the bones was readily effected in the following way: The diseased articulating surface and condyles of the humerus were made to project more freely backwards, and clear of everything else, by bending the forearm to nearly a right angle and drawing it forwards, the humerus being thrust in the contrary direction. I next placed the fine blade of my own saw in front of the bone, and cut off the condyles, with the expansion of the shaft immediately above them, from before backwards; the line of section verged upon the superior margin of the fossa for the olecranon. The extent of the part removed measured from above downwards about an inch and a quarter. I next freed the attachment of the brachius anticus from the coronoid process of the ulna, and having passed the blade of the saw in front of it and the neck of the radius, I cut them across by antero-posterior section, thus removing the diseased olecranon and head of the radius. The divided ends of the bones were carefully examined, and pronounced to be healthy; I clipped away with the scissors a good deal of disorganized tissues, and secured a few vessels which yielded blood, three on the outside, and the ulnar recurrent within; all were considerably enlarged from the previous persistence of disease in the part. The limb was next placed upon a splint at the right angle, and steadied above and below by a few turns of a bandage, the wound and parts adjacent being left exposed for the application of a few folds of linen steeped in spirit lotion. In this state the patient was removed to bed.

At 4 P. M., six hours after the operation, I proceeded to dress the limb. The man was free from pain, and there was no disturbance of the system; the bandages being taken off, and the limb remaining supported on the splint resting on its internal side, the glazed flaps were gently drawn together and made to meet in the transverse line, and retained so by three points of the interrupted suture; the longitudinal incisions were likewise approximated, and held so by a few stitches in their remote ends, the centre part being left open to permit any weeping or secretions to have a free escape; a few straps of adhesive plaster afforded additional support to the flaps. The wounds being so arranged, the arm was steadily lifted from the splint, retaining its somewhat more than rectangular position, and evenly rolled from the fingers to the shoulder; it was then placed in an evenly padded woolen case, accurately made for the purpose, with hinged sides, and the correct angle preserved. The forearm resting on its inner edge, or midway between pronation and supination, was steadied so by pads adapted to its inequalities; the sides, being elevated and fastened by a few straps of broad tape and buckles, steadied the entire immovably. By this mode of dressing, and this apparatus, the patient felt relieved from all apprehensions of displacing the bones; it lay evenly supported upon pillows, and somewhat raised at its lowest end. Wine, four ounces.

8 P. M.—No pain; no oozing of blood; no tension of the parts; ordered a full opiate, and to be repeated in the night if restlessnes should come on.

15th.—The patient slept nearly the entire night, and he had only to take the one draught. Pulse 92, soft and full; no headache; no pain in the wound; to have tea and toast, milk and eggs; spirits, four ounces; and beef tea with bread for dinner.

16th.—Report most favorable. To continue stimulants and food.

17th.—Slept all night after an anodyne; pulse 88, soft and full. Proceeded to dress the wound by letting down the sides of the box, and, slitting up the bandage from one end to the other, they were easily removed without lifting the arm from the support beneath; by this means all disturbance of the parts was prohibited; the flaps, at their junction in the transverse incision, were quite united, and the extremities of the lateral cuts were, in the same way, repaired; no additional swelling; no pouching of matter; a slight oozing of imperfect pus and serum has stained the dressings, and has had free room to escape, owing to the precautions already alluded to; lint smeared with simple unguent being laid over the wound, all bandages were now dispensed with; the pads were efficient in affording an equable support to every part of the limb; the sides of the box being closed up and fastened as before; stimulants and anodynes were freely administered.

19th.—Dressed the limb as at last report; wound looks most favorably; a free discharge of healthy pus; no swelling or tension of the parts; to continue stimulants.

28th.—Dressed the limb every alternate day since last report; and now the inordinate thickenings of the integuments and tissues around the joint, and constituting the flaps, is considerably diminished; the purplish livid discoloration has likewise nearly disappeared; the discharge is free and healthy from the interior, while granulations have sprung up, and appeared in the longitudinal wounds about their centre, where they had been suffered to remain apart for the special purpose of opposing union. On the whole, the parts are in a most satisfactory state, which, coupled with the total absence of all constitutional disturbance, promises most favorably for the issue. All ligatures have been cast off.

August 28th.—There is very little discharge now, and the case is rapidly progressing to cure; whatever is secreted comes from the interior of the wound, and escapes without obstacle through an aperture in the most depending part beneath; the soft parts around are much contracted, or, in other words, their hypertrophied condition is, in a very marked degree, reduced, the structures being consolidated, and the patient possesses the full power of moving the fingers as freely as ever. He has been sitting up in bed for the last ten days, and his general health is much improved. I removed the heavy box, and substituted a light pasteboard trough made after a similar form, and permitted the patient to walk about. From this period he continued daily to improve, and at length the discharge ceased altogether; bony union did not take place, yet the junction by fibro-ligamentous tissue was gaining rapidly thickness and solidity; at the same time the movemen of the fingers was quite perfect, and his grasp was as strong as before the operation; the forearm retained its motions of pronation and supination to the full extent; the only motion deficient was the full power of flexing the forearm when the limb was extended; I attribute this, in a great degree, to the emaciated state of the biceps muscle, which existed before the operation, for be it remembered that its attachment to the tubercle of the radius was not interfered with. Now to compensate for this loss of power I had recourse to the following expedient: A strong linen case was made to surround the false joint, and embrace the limb above and below it to an extent of from four to five inches; it was so formed as to sustain the forearm, at somewhat more than a right angle; it laced in front and was strengthened by pieces of whalebone placed exterior to the holes through which the cord passed; when this was adjusted the man could use a spade or shovel, and possessed great power with the limb.

The patient was now dismissed from hospital, and went to the country, and I lost sight of him for many months; on his return he called to see me; I found all the power and movements of the limb were quite perfect, with the exception of flexion of the forearm. I questioned him very closely as to the exercise which the limb had enjoyed, and was disappointed when I heard the linen case was seldom taken off, never by day, so that the muscles presiding over flexion of the limb were kept in restraint aud inactivity, and consequently were enfeebled, lowered in tone, with a wasting and diminution of all bulk. So satisfied was I of the true cause being explicable on this ground, that I retained the man in hospital for four or five weeks, removing all restraint, and permitting him to use the limb in every necessity, and moreover to work in the garden with the spade and shovel. At the end of this period the changes were marked; a perseverance in the exercise of the muscles developed their tone, tension, and volume, so that the patient actually regained the power of flexing the forearm on the arm. No doubt, the effect was slow, but it was sure; and as time passes so improvement will be superadded. At the present moment the man has had a limb preserved, well qualified for his numerous occupations.

Now with regard to incising the soft parts, many modes have been devised and recommended; but it need be of little moment to the dexterous surgeon, in ordinary cases, whether the flaps be single, double, or quadruple; the convenient exposure of the bones from behind is all that is required if there is much to be taken away, as in the case by which I have illustrated the operation. I am of opinion that the H incision, the mode inculcated, executed according to the rules which I have laid down, is the one most desirable.—By the internal incision the ulnar nerve can be drawn aside and taken away from injury. As to the cutting of the bones, I am of opinion that it can be most readily achieved by at once cutting through the insertion of the triceps and the lateral ligaments, and then disarticulating; all the remaining muscular fibres should be detached, and the osseous parts can be cut through with great facility from before backwards by aid of the saw which I have figured in this journal for February of the present year; the fine blade of the instrument is readily placed in front of the bones, the flaps do not interfere with its working, and the rapidity with which it cuts is most pleasing to the operator; but, above all, the section is perfectly smooth and even; this latter perfection is borne ample testimony to by the numerous specimens in my collection, and which I have excised from the living subject with it.

Not only does the early disarticulation facilitate the after-steps of the operation, but it is of incalculable advantage, as at once revealing the amount of disease, and as to how far these bones may need resection. The bones removed in the case which I have detailed afford so beautiful a specimen that I cannot forbear giving an engraving of them. It will at once be seen how completely limited to the joint the caries was, and how a cavity existed in the end of the humerus, which necessitated the section of the bone a little above the condyles. A small abscess will likewise be seen to exist in the olecranon process of the ulna. The head of the radius exhibits in its centre and on its outside a small remnant of its cartilage of incrustation, while the worm-eaten appearance of the bone around is very characteristic of the affection.

As to the after-treatment of the limb, all arteries should be tied, particularly the posterior ulnar recurrent; if not secured, this vessel will surely bleed after the patient becomes warm in bed, and give a great deal of trouble by requiring the flaps to be again separated to permit of its being ligatured. Roux gives one solitary instance where a patient under his care died of secondary hemorrhage. The limb should be steadied in the box with falling sides, such as I have described, and maintained as steady as possible for many days; by this gentle manipulation the part may be induced not to resent the violence offered to it by any destructive burst of inflammation. After the flaps have become united, and consolidation of the parts within has progressed to firmness, about the end of the fourth or fifth week, very small and passive motion may be commenced, so that, by carefully apportioning exercise to the repaired and condensed structures, the limb may be restored, efficient and useful for all the purposes of life. Sometimes the union between the bones may be slow in consolidation; it was so in the instance which I have recorded, yet after the lapse of some months, it was sufficiently firm for all the necessities of manual labor. The surgeon, I say, then, need not be disheartened by the tediousness of recovery; in the end it will come. It was so likewise in one of Moreau's cases, who writes thus: "The flexion of the forearm upon the arm is strong, firm, and steady; it was a long time before the movement was regained; when he wanted to bend the arm, the forearm shook and fell in toward the inner side, but he has got the better of that of late, and now this motion is free and correct." Further on he mentions, in relation to this case: "I must not forget to state, that this man has now the use of his arm so completely that he uses it in thrashing in the barn, holding the plough, &c."

I shall append to these remarks on excision of the elbow-joint, the impressive and simple words of Moreau: "If these things seem to be incredible, they may be easily brought to the test of experiment. I am firmly of opinion, that, in similar circumstances, the issue will be the same."—*Dublin Quart. Journ. of Med. Science*, Nov., 1855.

